# Public Baths with Special Appliances

**Published:** 1901-04-20

**Authors:** 


### PUBLIC BATHS WITH SPECIAL APPLIANCES.

Bath, Hot Mineral Springs.—The city of Bath has a very fine bathing establishment, where, in addition to the use of the Bath waters, many modern hydro-therapeutic methods are available, among which are the following:—Thermal vapour baths, thermalsoolbader (Nauheim system), the Aix massage douche, needle douche, Scottish and Vichy douches, medicated baths, Cadet's sitz bath, pulverised water inhalation rooms, and complete arrangements for bouillon, spray, shower, dry hot-air baths by electricity.

Principal hotels: Grand Pump Room Hotel, York House Hotel.

Buxton Public Baths.—The bathing establishment at Buxton, in addition to the use of Buxton waters, possesses the necessary appliances for every kind of bath, douche, and massage.

Hotels: Crescent, Eagle, George, Grove, Lee Wood, Midland, Old Hall, Palace, Royal, Savoy, Shakespeare, St. Ann's.

Droitwich, The Brine Baths.—Muriated saline baths, the strongest in Europe, 10 to 12 times as strong in salt as sea water. Temperature as required. Employed in the form of soak and needle baths, and in large swimming baths maintained at a temperature of 85° Fahr.

Hotels: Raven and Worcestershire.

Railway station: Droitwich, Great Western Railway.

Communications to the Secretary, Brine Baths, Droitwich.

Harrogate Royal and Victoria Baths, Yorkshire.—The Corporation of Harrogate has lately spent a very large amount of money in setting up a complete bath establishment, at which, besides the internal use of the waters, all the appliances for complete hydro-therapeutic treatment are made available, as well as other modern modes of cure. The following are the methods which are in use: Strong or mild sulphur baths (supplied from the springs), vapour baths, Harrogate massage douche, needle and shower douche (combined baths), running or still sitz baths, Vichy douche, ascending and descending douche, fresh-water baths, dry massage, "Berthe" dry and moist heat, Nauheim, hot-air baths, chromopathic sun bath, Dr. Finsen's apparatus for the treatment of lupus, &c. Communications to Manager, the Royal Baths, Harrogate.

Hotels: Majestic, Clarendon, George, Granby, Prince of Wales', Queen, Royal, Somerset.

Llangammarch Wells Barium Springs, R.S.O., Breconshire.—Medical officer: Dr. W. Black Jones. Principal features: Nauheim and Oertel's methods for the treatment of cardiac disease. Course of treatment: Four to six weeks. Railway station, Llangammarch Wells, London and North-Western Railway, Central Wales branch.

Principal hotel: Lake Hotel, Llangammarch Wells.

Sidmouth Baths, Sidmouth, Devon.—Situated at the south-east corner of Devonshire, and protected by a semicircle of hills on the north and east. Nauheim treatment carried out under climatic conditions that are favourable all the year. Aix douche, massage, brine, and other baths. Nearest railway station, Sidmouth.

Principal hotels: Knowle, Bedford, York.

Strathpeffer, Highland Sulphur Spa, Ross-shire.—Sulphur and chalybeate waters. Sulphur, mud, Russian, pine and needle baths, douches (Nauheim treatment). Season, April to October.

Hotels: The Ben Wyvis, Strathpeffer, and Spa.

Railway station: Strathpeffer Highland Railway.

Woodhall Spa, Lincolnshire.—Special feature: Saline bromo-iodine waters, rich in iodine. A large new bath-house has been erected with every accommodation for baths, douches, inhalations, pulverisations, &c.

Hotels: Eagle Lodge and Victoria.

New Hospital for St. Andrews.—Plans have been prepared and accepted for the new hospital at St. Andrews, which is to be erected in memory of Lieutenant Tate. The site is a favourable one, in Abbey Park. On the ground floor is to be situated the Lieutenant Tate Memorial Ward with two beds for male patients. On the same floor there is to be another male ward for four beds and a female ward for four beds. Upstairs there is to be a small ward for females and a children's ward. Offices and administration apartments will be distributed over the two floors, whilst the kitchen is in a separate block, connected to the hospital by corridors.

